# Blockchain-Based Lightweight Trust Management in Mobile Ad-Hoc Networks

**DOI:** 10.3390/s20030698

**Published:** 2020-01-27

**Authors:** May Thura Lwin, Jinhyuk Yim, Young-Bae Ko

**Affiliations:** 1 Department of Computer Engineering, Ajou University, Suwon 16499, Korea; maythura@ajou.ac.kr; 2 Electronics and Telecommunication Research Institute, Daejeon 34129, Korea; jhyim@etri.re.kr

**Keywords:** blockchain, distributed trust, mobile ad-hoc network (MANET), optimized link state routing protocol (OLSR)

## Abstract

As a trending and interesting research topic, in recent years, researchers have been adopting the blockchain in the wireless ad-hoc environment. Owing to its strong characteristics, such as consensus, immutability, finality, and provenance, the blockchain is utilized not only as a secure data storage for critical data but also as a platform that facilitates the trustless exchange of data between independent parties. However, the main challenge of blockchain application in an ad-hoc network is which kind of nodes should be involved in the validation process and how to adopt the heavy computational complexity of block validation appropriately while maintaining the genuine characteristics of a blockchain. In this paper, we propose the blockchain-based trust management system with a lightweight consensus algorithm in a mobile ad-hoc network (MANET). The proposed scheme provides the distributed trust framework for routing nodes in MANETs that is tamper-proof via blockchain. The optimized link state routing protocol (OLSR) is exploited as a representative protocol to embed the blockchain concept in MANETs. As a securely distributed and trusted platform, blockchain solves most of the security issues in the OLSR, in which every node is performing the security operation individually and in a repetitive manner. Additionally, using predefined principles, the routing nodes in the proposed scheme can collaborate to defend themselves from the attackers in the network. The experimental results show that the proposed consensus algorithm is suitable to be used in the resource-hungry MANET with reduced validation time and less overhead. Meanwhile, the attack detection overhead and time also decrease because the repetitivity of the process is reduced while providing a scalable and distributed trust among the routing nodes.

## 1. Introduction

Unlike conventional networks, mobile ad-hoc networks (MANETs) are cooperative networks composed of mobile nodes. Without any fixed infrastructure or centralized administration [1], the nodes randomly move and communicate with each other both directly via wireless connections and indirectly via communication with other nodes outside the wireless range through relay nodes. This mode of communication induces the multi-hop phenomenon [2], where intermediate nodes act as routers for relaying packets to destination nodes. Consequently, several routing protocols for MANETs have emerged, including optimized link state routing (OLSR), ad-hoc on-demand distance vector routing (AODV), and dynamic source routing (DSR). Based on these routing protocols, the nodes themselves choose a routing path to the destination and forward packets [3,4].

Based on their flexibility and robustness, regardless of geographic location or proximity to infrastructure [5], MANETs are applicable to various interactive application scenarios, including military battlefields, commercial sectors, and other civilian environments. In military scenarios [6], while MANETs can provide information network technology for soldiers and vehicles through scattered devices on the battlefield, an enemy can compromise devices and make the network vulnerable, allowing them to disseminate malicious information, discard sensitive information in multi-hop scenarios, or extract critical data. Additionally, in after-war scenarios, an enemy can continue to scatter devices in an attempt to obstruct recovery operations or coordinate sudden attacks. MANETs are also widely applied in the commercial sector, as well as in emergency and rescue operations following natural disasters. During emergency and rescue operations, MANETs are also prone to vulnerabilities. For example, when multiple countries cooperate to recover from natural disasters, the data that some of the countries prefer to secure as private and sensitive could be exposed through MANETs [7].

Securing communications in MANETs is one of the biggest challenges for system architects. There are a variety of attacks that can occur in every network layer (the attacks that MANETs are vulnerable to are classified in Table 1). Although it is desirable to introduce effective security solutions that can reveal attackers in a network and there are several existing MANET security solutions, attack prevention is even more desirable. To minimize malicious actions, it is necessary to introduce an effective trust system in MANETs to achieve various system objectives, including reliability, scalability, and availability [8].

To introduce trust into communication networks under critical conditions, system designers should consider various perspectives (e.g., node heterogeneity, rapid changes in network topology, a lack of predefined trust relationships, and the resource-constrained environment of mobile nodes [9]). In MANETs, trust management has been introduced in various forms, including trust establishment [10], trust updating, and trustworthiness [8]. Some researchers have introduced an authentication mechanism [11] to ensure links are safe prior to communicating within the network. However, because MANETs are continuously changing [12], the owner of a secret key could be a malicious entity, even if there are no malicious outsiders with unauthorized identities. Furthermore, it is difficult to control the authentication process through a single node. Generally, trust operations are limited to local access, which introduces the problems of incompleteness and vagueness. Hence, it is critical to reconfigure networks seamlessly and consistently.

In this paper, we propose a blockchain-based trust establishment system that provides distributed, consistent, and tamper-proof trust to nodes in MANETs. Blockchain as a potential solution for trust management, it has been actively researched in various fields, including wireless networks [13] and the internet-of-things (IoT). To take advantage of the decentralized nature of blockchain technology, one must consider the limited resources of MANETs when designing a trust system. For example, implementing a blockchain implementing a blockchain with proof-of-work (PoW), the complex computational consensus mechanism and long validation time in MANET nodes without any centralized party would be highly impractical. Therefore, we propose a lightweight consensus algorithm for trust management in MANETs called delegated proof-of-trust (DPoT), which achieves reasonable validation times based on peer-to-peer (p2p) concepts.

Although various attacks can occur in any security layer of a MANET, in this study, we decided to focus on evaluating the trust of a node based on routing issues, which occur in the network layer and represent a critical research issue in dynamic MANET environments. OLSR [14] was selected as a representative routing protocol for implementing blockchain operations since it is one of the proactive protocol which maintains the neighbor information. The proposed system is a trust management system using a policy-based and reputation-based trust management framework. Therefore, a policy-based mechanism that can generate binary results according to whether or not a node is trusted is defined such that trust evaluation (reputation-based evaluation) can be performed based on binary results. We implemented denial contradictions with a fictitious node mechanism (DCFM) [15] as a representative detection mechanism for identifying malicious attackers and trusted nodes as this is one of the effective schemes that was introduced recently.

Moreover, this paper considers to reduce the unnecessary bandwidth and energy usage by introducing the collaborative detection process based on predefined principles. Most security solutions in OLSR, including DCFM, force nodes in the network to perform excessive operations based on frequently updated topology information, which is periodically exchanged and sometimes contains additional messages, which incur unnecessary bandwidth and energy usage. Furthermore, nodes are suspicious of each other since they are on their own without any authentication process. This gives attackers a chance to compromise networks easily and manipulate residential nodes repeatedly which makes the overall network efficiency is decreased.

The main contributions of this paper can be summarized as follows.

- We design a trust evaluation system that can fulfill the objectives of MANETs based on blockchain technology.
- The blockchain implementation is described based on a series of steps, transaction validation, block configuration, block validation, and block chaining and maintenance in MANETs. Furthermore, a suitable consensus mechanism for MANETs called DPoT is successfully embedded in OLSR for the validation of blockchains.
- We designate several principles to determine if nodes can implement a particular security mechanism to mitigate attacks in a collaborative manner to reduce energy consumption and network vulnerability.

The remainder of this paper is organized as follows. In Section 2, we discuss background information and related works. In Section 3, we introduce the proposed method. In Section 4, we describe our simulation environment and test results. Our final conclusions are discussed in Section 5.

## 2. Background and Related Work

### 2.1. Overview of OLSR

**Figure 1:**
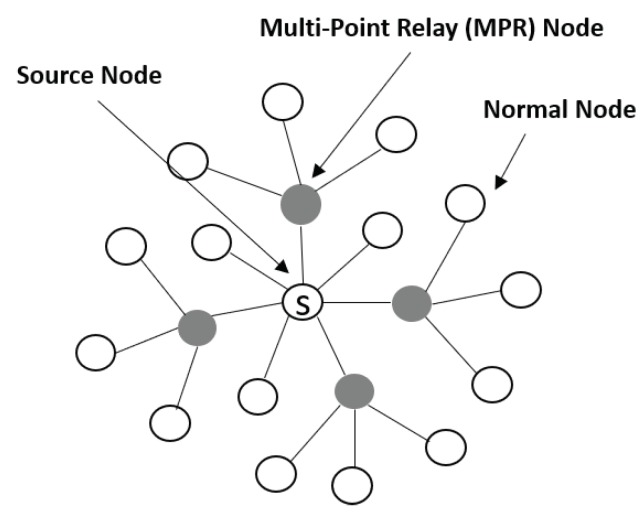
How MANET Nodes Work with OLSR.

The OLSR [14] protocol is a proactive routing protocol for MANETs. It is an optimized version of the classical link state routing protocol that reduces control message overhead. The nodes in a MANET select an MPR node to provide maximum coverage for their two-hop neighbors and reduce the number of control messages. With a minimum number of MPR nodes, a node can reach its neighbors through a small number of transmissions without any duplicated messages, as shown in Figure 1. MPR nodes are responsible for relaying both control messages and data messages.

**Figure 2:**
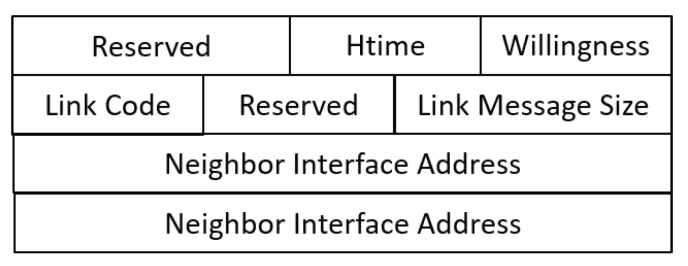
HELLO Message Format (RFC 3626–OLSR).

**Figure 3:**
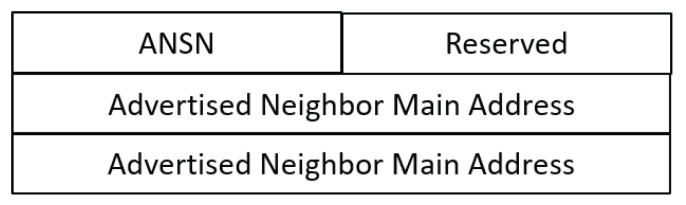
TC Message Format (RFC 3626–OLSR).

The OLSR node maintains the topology information for the network through periodic exchanges of control messages. There are two main types of messages that describe the topology information of the network, namely the HELLO message (described in Figure 2) and topology control (TC) message (described in Figure 3). Every node includes its neighborhood information (one-hop neighbor nodes) in a HELLO message and broadcasts it to all one-hop neighbors. Subsequently, each node collects the topology information of the network until reaching two-hop range. TC messages are advertised by MPR nodes. These messages include a list of MPR selectors that have chosen the broadcasting nodes as their MPR nodes. Every routing node acquires the network topology by utilizing hello and TC messages. Finally, the shortest route to a destination node is calculated and data packets are forwarded efficiently.

### 2.2. DCFM: Representative Attack Mitigation Scheme in OLSR

DCFM is a representative scheme that was adopted in the proposed scheme. Before detailing DCFM, we will discuss node isolation attacks (NIAs), which are the main attacks that are explicitly addressed by DCFM. NIAs, which were first defined by Kannhavong et al. [16] in 2006, are a type of DoS attack against the OLSR. As the name suggests, the objective of this attack is to isolate a targeted victim from the network. In such attacks, the attacker takes advantage of the fact that OLSR routing nodes find the MPR nodes with the maximum coverage among their neighbors. Specifically, the attacker node prevents a victim from receiving control messages from its neighbors.

As a starting point, the attacker positions itself within the broadcast range of the victim and identifies its two-hop neighbors by exchanging hello messages with the victim. It then produces a fake hello message indicating that it is a one-hop neighbor of the victim’s two-hop neighbors. Consequently, the victim will choose the attacker as its sole MPR based on the routing protocol, which requires a minimal set of MPRs. Therefore, the attacking node is the only node that can forward link information from the victim and it simply discards the victim’s messages. As a result, other nodes cannot receive any information from the victim, so will be removed from the network topologies of any other nodes.

The symbols used to explain DCFM are defined as follows.

- *N* is the set of all nodes in the network.
- *n*; x∈N are the victim and the attacker nodes, respectively.
- $x_{1}$ is a fictitious node advertised by *x*.
- $HOP_{1}\left(n\right)\subset N$ is the set of all one-hop neighbors of *n*.
- $HOP_{2}\left(n\right)\subset N$ is the set of all two-hop neighbors of *n*.
- $MPR\left(n\right)\subseteq HOP_{1}\left(n\right)$ is the set of one-hop nodes of *n* who have appointed *v* as their MPR.
- $MPR^{\prime }\left(n\right)\subseteq HOP_{1}\left(n\right)$ is the set of one-hop nodes who were selected by *n* as MPRs.

**Figure 4:**
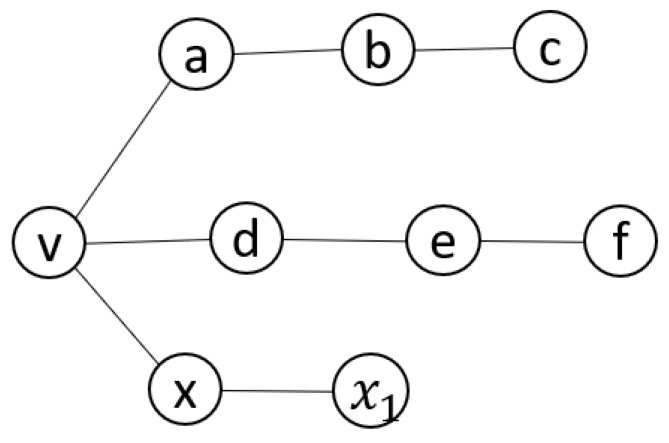
Sample Network with an NIA.

First, when node x sends a HELLO message containing HOP1(x), n should check if HOP1(x)∩HOP1(n)=ϕ. For example, in Figure 4, HOP1(v) = [a, d] and x is attempting to isolate node v. Therefore, it sends a fake hello message claiming to know all two-hop neighbors of v ($HOP_{1}\left(x\right)$ = [v, b, e, $x_{1}$]). The reason why x excludes v’s one-hop neighbors is that v could determine if x is an attacker by checking the hello messages of its one-hop neighbors, allowing it to mark x as a suspicious node.

However, x declares that v is its one hop neighbor, meaning all one-hop neighbors of v are two-hop neighbors of x, so it can be assumed that x will choose v as its MPR to cover all nodes in $HOP_{1}\left(v\right)$. Therefore, x cannot be identified as malicious, so additional contradictions must be identified based on a second rule.

For each node y mentioned in a HELLO message, n should check if there is a node $z\in HOP_{1}\left(y\right)$ such that (a) z is not included in $HOP_{1}\left(n\right)$, (b) z is located three hops away from n, or (c) there is a specific node in MPR(x) for reaching z. For example, in Figure 3, according to x’s hello message, b and e are one-hop neighbors of x. Consequently, b and e are only one-hop neighbors of c and f, respectively. This means c and f are three hops away from v. Therefore, x should appoint b and e as its MPRs for covering c and f, respectively. Otherwise, it is determined that x is an adversary.

The final rule is that node n should check every HELLO message containing its neighbors if the sender is suspicious. These rules should be applied sequentially. In this scheme, all nodes in the network perform these processes repetitively in the hello interval and TC interval whenever a hello message is suspicious. Because nodes perform the attack mitigation process individually without trust considerations for other nodes, in the case where there is no attacker in the network, it is wasteful for every node to be protective all the time. Furthermore, some nodes in the network must add virtual nodes under certain conditions when an attacker is intelligent and makes negligible changes to the control messages exchanged.

### 2.3. Trust Management in MANETs

In the dynamic environment of a MANET, trust management plays an essential role [8]. Therefore, several studies have developed various approaches, such as reputation-based frameworks, trust establishment frameworks, and policy-based trust management. In reputation-based trust management [17], trust values are calculated by aggregating and distributing reputation among residents. In this scheme, a destination node rates the reputation values of its upstream nodes by checking the packets delivered and acknowledges the source node. Based on the occurrence of transactions, each node that participated in the message transmission process maintains the trust values of other participants in their distributed routing table. However, trust values are not verified, meaning if the rating node is malicious, all nodes that participate in message transmission can be manipulated in this scheme. Additionally, the rating process occurs after packet transmission, meaning sender nodes cannot be sure of the trust values of their relay nodes.

Some researchers have argued regarding the limitations of reputation-based systems, which include vagueness, complexity, and inaccurate characterization [18]. Some have proposed policy-based trust management, where logical rules are used to distinguish trusted entities. In [18], the authors combined policy-based and reputation-based trust systems to create an integrated system that enhances the security and flexibility of other solutions. Trust is necessary for both service-requesting nodes and service-providing nodes. A requested service is evaluated based on control policies, while the provider must also verify that its reputation is sufficiently high to satisfy the request. Although this method meets the needs of trust management in MANETs by combining two mechanisms, it heavily depends on prior information, rather than dynamic trust management.

### 2.4. Blockchain-Based Trust Management

Blockchain technology has become popular based on its strong security. This technology emerged from Bitcoin in 2008. Based on its strong security and decentralized management, it has been applied to various types of monetary transactions. In summary, several transactions are performed in a transaction pool. Several nodes attempt to create blocks of unspent transactions to perform a mining process, resulting in a proof-of-work (PoW), which indicates that a block has been created, accepted, and chained into the existing blockchain. After chaining a block into the chain, it is difficult to change information in the block because new blocks can be chained only when a miner obtains the correct nonce that can generate a valid hash value. Hash values are generated based on the nonce that is mined and the previous block hash.

Owing to its security, potential applications of blockchain in various areas technology has been widely researched. Blockchain-related research on trust management for wireless and ad-hoc network security has recently been published. In [13], a trusted routing scheme based on blockchain technology and reinforcement learning was introduced for wireless sensor networks. In this work, routing activities were stored in a blockchain as transactions for the purpose of non-repudiation while reinforcement learning helped routing nodes choose trusted relay nodes based on information stored in the blockchain. Two types of nodes were defined: validators, which maintain the blockchain; and minions, which track routing information in the network. Validators performed blockchain validation by using proof-of-authority (PoA) to eliminate the resource-consuming tasks in PoW. However, based on the influence of the centralized and predefined validator concept, this method seems to be inappropriate for decentralized MANETs.

Goka et al. [19] introduced a distributed management system for trust and reward (DMTR) in MANETs. The DMTR utilizes a blockchain to enhance the conventional reputation system, which computes node reputation values based on node behavior and a price-based system, where a sender node gives points to relay nodes. General nodes report information regarding neighboring nodes in the network to specific mining nodes and the mining nodes manage the blockchain. The mining nodes collaborate to create a single target block by eliminating the application of PoW in MANETs. In this work, although the authors have proposed a distributed management system, blockchain management relied on a centralized authority with some predefined trusted nodes because blockchain processes are too heavy to be handled by routing nodes in MANETs.

A blockchain-based decentralized trust management scheme was also introduced for vehicular networks [20]. Based on the high mobility and dynamic nature of vehicles, vehicular networks require a trustful environment, similar to MANETs. In this paper, the authors have introduced blockchain technology combined with PoW and proof-of-stake (PoS) to regulate the benefits of both mechanisms. Vehicles performed a rating process for other vehicles, while roadside units (RSUs) verified and maintained the TVs in the blockchain network. Because blockchaining was conducted by RSUs, this scheme could be considered as a decentralized system for vehicular networks. However, for completely distributed MANETs without centralization, the application of a more decentralized and lightweight blockchain for trust management is still an important research topic.

## 3. Proposed Solution

### 3.1. Design Objectives

1. Decentralization: Based on the flexible nature of MANETs, where nodes continuously enter and leave a network without administrative action, trust evaluation for resident nodes is an essential component. However, it is impractical to assess nodes within multi-hop distance using only a centralized node or set of nodes. Therefore, decentralized trust evaluation is crucial for MANETs, meaning the nodes in a MANET should perform mutual assessment that is decentralized and straightforward.
2. Availability & Consistency: For decentralized systems, such as MANETs, the availability of data is an important factor that must be considered. Each node should have trust values (TVs) for its relay nodes, even when they exist at multi-hop distance, so it can select the optimal nodes for forwarding data. Additionally, the data accessed by all nodes should be consistent for each node.
3. Tamper-Proofing: Based on some of the aforementioned design goals, such as decentralization and availability, all available information will be accessed by all nodes in the network. Even with reliable support from the proposed scheme, critical information should not be tampered with by malicious nodes in the network. In other words, the system should be resilient to a small set of collaborative adversaries.
4. Lightweight performance: Based on strong security goals, it is important that any trust solution provide reasonable performance, even with the complex computations required for authentication. However, for MANETs with limited resources, it is desirable to use a straightforward and effective solution, rather than a strongly secure, but complicated mechanism.
5. Efficiency of security model in MANETs: To the best of our knowledge, most trust solutions require repetitive processes to be performed based on the dynamic nature of MANETs. Moreover, a network is vulnerable to repeated attacks from the same attacker when there is no collaboration between individual nodes. Therefore, a trust solution with greater efficiency must be developed for MANETs.

### 3.2. Blockchain-Based Lightweight Trust Management in MANETs

**Figure 5:**
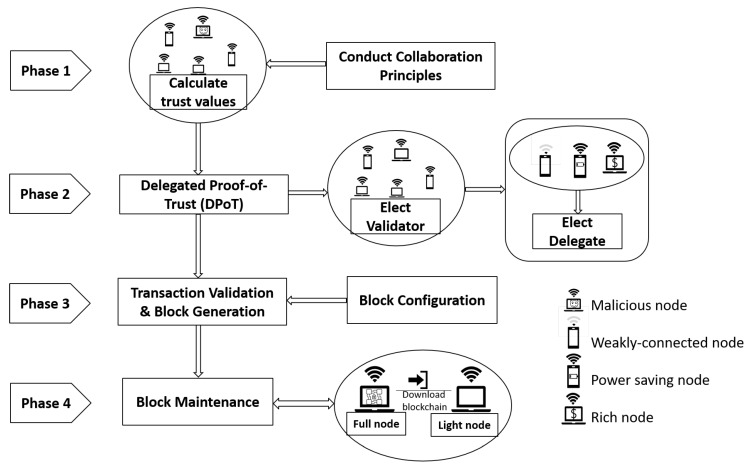
System Design for a Lightweight Trust Blockchain.

With the above design goals in mind, we adopted a blockchain architecture to manage trust evaluation and maintenance in a MANET environment. Specifically, we implemented a public blockchain architecture to eliminate the intensive resource consumption and long validation times of current blockchain technology in a dynamic and latency-sensitive environment. Based on the requirements of MANETs, the proposed blockchain-based distributed trust establishing system can be divided into the components presented in Figure 5.

#### 3.2.1. Phase 1—Trust Value Calculation

Because the objective of this work was to develop a distributed trust system for improving network reliability and scalability, we focus on how to construct a trusted network, rather than how trust should be calculated. Furthermore, to reduce vulnerability to repeated attacks by the same attacker, whenever a node detects an adversary in its vicinity, the attacker information is distributed through the network. A variety of detection schemes and trust models will work in the proposed scheme, but the DCFM [15] was adopted as a representative scheme that is applicable to the solution presented in this paper. All nodes in the network follow the DCFM rules to detect malicious neighbors. As discussed above, the DCFM algorithm detects adversaries by analyzing topology information obtained from neighboring nodes. If there are any contradictions in the received information, the sender node is identified as a malicious node and receives a large penalty from the checking nodes. As soon as a particular node is identified as an adversary, that node is effectively removed from the network by sharing its information across the network via blockchain technology.

To specify explicit and fair rewards and penalties for residential nodes, including adversaries in MANETs, we adopt the additive increase/multiplicative decrease (AIMD) [21] scheme to control the TV of each node. As the name implies, the TVs of nodes will be added and multiplied based on α and β factors, respectively, as shown in Equation (1). In the DCFM detection strategy, an identified attacker’s TV will be multiplied by a β value of -1, which represents the worst penalty in the network. The information of nodes with negative TV values is distributed through the network so every resident can eliminate such nodes from their connections (the validation of that information (block transaction) will be discussed in a later section). Negative trust value means that the node is banished from the network since the trust value for the nodes in this network is only between 0 and 1. However, for nodes that do not intentionally attack neighboring nodes, but are attempting to be selfish, the β value will be different, as described in Equation (2). In contrast, nodes that behave appropriately will gain increasing TVs by adding α values. For the sake of fairness of decisions on the trust level, the MPR node should have a greater value compared to other nodes. Therefore, α is adjusted based on Equation (3). In the case of the MPR node, if it has been the MPR once according to the evaluating node, its α value will be $\frac{1}{1+1}=\frac{1}{2}=0.5$. If it contributes two times, this value will be $\frac{2}{3}=0.67$, and so on. For honest nodes that are not MPRs, α will be set to 0.1 and such nodes will accumulate high TVs over time. However, every node can have the trust value, ‘’1” as the highest. The initial TV value for every node in the network is zero.

- TV = Trust Value, 0≤TV≤1
- α = additive factor
- β = multiplicative factor
- $\sum_{k=1}^{n-1}M_{i}j^{k}$ = number at which node *i* chooses node *j* as its MPR node to forward packets for *k* iterations (starting from when *j* begins to have a connection with *i* to when *i* calculates *j*’s TV

(1) $$TV=\left\{\begin{array}{ll} TV*\beta , & \;\mathrm{if}\;a\;\mathrm{node}\;\mathrm{misbehaves}.\\ TV+\alpha , & \;\mathrm{otherwise}. \end{array}\right.$$

(2) $$\beta =\left\{\begin{array}{ll} -1, & \;\mathrm{if}\;a\;\mathrm{node}\;\mathrm{is}\;\mathrm{an}\;\mathrm{attacker}\;\mathrm{according}\;\mathrm{to}\;\mathrm{DCFM}.\\ 0.5, & \;\mathrm{otherwise}. \end{array}\right.$$

(3) $$\alpha =\left\{\begin{array}{ll} max\left[\frac{\sum_{k=1}^{n-1}M_{i}j^{k}}{\sum_{k=1}^{n-1}M_{i}j^{k}+1},0.1\right], & \;\mathrm{if}\;a\;\mathrm{node}\;\mathrm{is}\;\mathrm{an}\;\mathrm{MPR}\;\mathrm{node}.\\ 0.1, & \;\mathrm{otherwise}. \end{array}\right.$$

Furthermore, with the goal of making the proposed system more efficient, we introduce a cooperative mechanism for our security solution. Although cooperative networking has been considered previously for MANETs [22], in most of the security mechanisms available for proactive routing protocols, nodes perform individual detection processes. In DCFM, detection occurs in every hello interval. However, in our solution, the detection interval is decreased according to the number of neighbors in the node vicinity. By taking advantage of the synergistic effects of nearby neighboring nodes, the detection interval can be increased. Nodes that fulfill the following principles can perform collaborative detection instead of working individually.

- $N_{1}$, $N_{2}$, $N_{3}$, … = residential nodes in the network,
- $S_{1}$, $S_{2}$, … = number of nodes in the neighbor set of $N_{1}$, $N_{2}$, …, respectively,
- $I_{0}$ = the initial checking interval (same as the hello interval, typically 2 s),
- $I_{1}$ = the increased detection interval when collaboration can be performed,
- *f* = multiplicative factor used for increasing the detection interval

1. At least three nodes should have mutual connections (i.e., nodes $N_{1}$, $N_{2}$, and $N_{3}$ are exchanging control messages with each other). A collaborating node should not be a node that just joined the network.
2. For determining the length of the interval, a node should consider the number of nodes involved in the hello messages with other neighbors (see Equations (4) and (5) for the calculation of a new detection interval).
  (4) $$f=min[\;S_{1},\;S_{2},\;S_{3}\;].$$
  (5) $$I_{1}=I_{0}*f.$$
3. After determining the new detection interval, task assignment will be performed based on the order of node addresses (e.g., IP addresses of nodes). The assigned order increases from the smallest to the largest address. For example, among three nodes $N_{1},N_{2}$, and $N_{3}$, the detection-duty order = [$N_{1},N_{2},N_{3}$].

As discussed in Principle 1, for realizing fully protective and collaborative detection, at least three nodes must work together so that even if node $N_{1}$ is being manipulated by an attacker and $N_{2}$ is taking a break from detection duty, node $N_{3}$, which is connected to both nodes, can still detect the attacker. However, for complete security, new nodes should not be included in the collaboration process. A node should only perform detection in this situation if it has been contributing to the network for a specific amount of time and earned a reasonably high TV.

The new interval for a node is based on the number of nodes mentioned in the hello messages of its neighbors. According to the first principle, the multiplicative factor *f* will start from a minimum value of two, which is the minimal count of required collaborators in addition to the target node itself. After multiplying the value of *f* by the initial interval $I_{0}$, the resulting value $I_{1}$ will be utilized as the new detection interval. Therefore, it can be concluded that the greater the minimum number of neighbors, the longer the detection interval for nodes.

For a value of $I_{1}$, collaborating nodes should determine the task assignment order in the same manner as a peer-to-peer network. In this context, the network may be vulnerable to malicious attacks based on misunderstandings between nodes. Task assignment should be performed based on a global value that is unique for every node. Therefore, we set the node address to a value suitable for determining the task assignment order. The node with the smallest address number is responsible for the first interval and the node with the greatest address number is responsible for the final interval.

**Figure 6:**
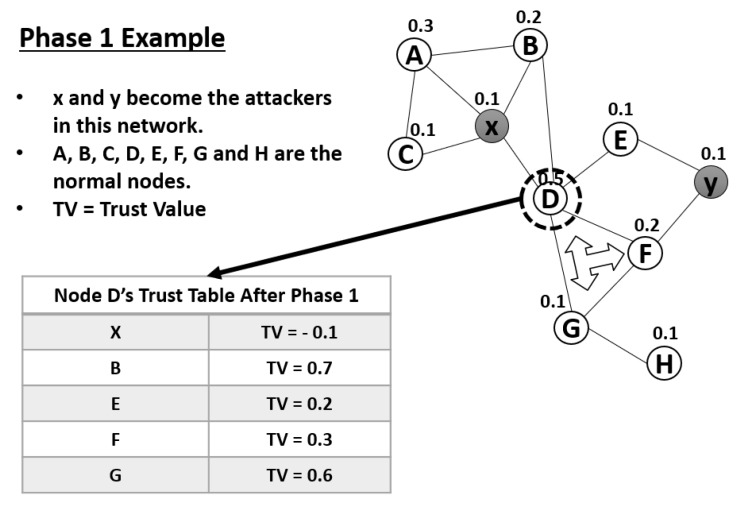
Demonstration of Phase 1.

In Figure 6, we demonstrate phase 1 with two attackers in MANET. After phase 1 is completed, the trust tables of the nodes will be updated. For example, in node D’s trust table, all of its neighbors will be evaluated according to the equations presented above. Because node X is an attacker, its old value of 0.1 will be updated via multiplication by −1. Nodes E and F receive an α value 0.1, while nodes B and G receive values of 0.5 because they are MPR nodes for node D. Furthermore, in this network, nodes D, G, and F can perform collaborative detection according to the principles outlined above.

#### 3.2.2. Phase 2—Consensus Algorithm (Delegated Proof-of-Trust)

Calculated trust information is included in blocks such that every node in a network can consistently access the information simultaneously. In a MANET environment, because there is no centralized party that handles blockchain processes, the consensus process must be performed by routing nodes in a distributed manner. Delegated proof-of-trust (DPoT) is introduced to compensate for the downsides of other consensus mechanisms by considering MANET characteristics. For example, proof-of-work (PoW), which uses computationally complex operations to perform validation, is not a suitable choice when energy consumption is a primary concern. Although another candidate mechanism called proof-of-stake (PoS) works well in resource-constrained environments, the “nothing-at-stake” problem cannot be ignored. Because this algorithm uses predefined tokens (stakes) to identify validators, it is impractical to apply it to a temporary ad hoc network. Furthermore, its biggest limitation is that it gives the richest nodes in the network get the greatest chance to become validators, meaning it lacks fairness. Therefore, we propose DPoT as a new consensus algorithm for dynamic and resource-hungry environments.

##### Validator Election

The nodes with the highest TVs are the candidates to become validators in the network. To determine which node will be the block creator, we adopt the bully election [23] strategy, which is one of the common election algorithms used in distributed environments. It is called bully election because the nodes with the highest identification numbers make other nodes accept them as coordinators. A node that intends to become the leader contacts any other nodes with higher priorities. If there is a reply from any of those nodes, it gives up on becoming the coordinator. Otherwise, it becomes the coordinator in the network. When the highest priority node directly claims the coordinator role, the communication overhead is the lowest, meaning this is the best scenario. Therefore, this algorithm is appropriate for a MANET environment.

Similarly, in a MANET blockchain, only the node with the highest TV can become the validator. There is a threshold value θ in the network that determines if a specific node is sufficiently trustworthy to become a validator node. Unlike in bully election, a node cannot claim to be a trustful node on its own, meaning it requires a neighboring node. If node *i* has a neighboring node *j* with a TV above the threshold, it sends a claim message (i, j, TV-Claim, one-hop-count)$prKey_{i}$, where *i* is the follower node of *j*, *j* is the validator node claimed by *i* and trust value and one-hop neighbor count of *j* is put in TV-Claim and one-hop-count, respectively. $prKey_{i}$ is the private key of *i*, which signs the claim message. Similarly, every node with neighbors with TVs above the threshold can broadcast a claim message to the entire network by piggybacking on a TC message. If the TV of node *j* is the highest and there are no malicious claims on nodes *i* and *j* from other nodes, *j* becomes the validator. In the claim message, one-hop neighbor count is added to avoid the situation in which two or more nodes have the same trust value and cannot be concluded which node should be selected. Based on the count, the node which has more one-hop neighbors could have more chance to be validator. Here, node *i* sacrifices its energy to broadcast a claim message for node *j*. Because a MANET is a resource-hungry environment, there should also be a reward for the voting node *i* (possible rewards for node *i* will be discussed in the following subsection).

##### Delegation Process

Based to the dynamic and limited features of MANETs, even though a node is chosen as the validator, there could be the time when it cannot do the block generation process. Therefore, we consider a delegation process for the validator node that is influenced by the delegated proof-of-stake (DPoS) algorithm, where the validator from the PoS mechanism can vote on whether or not other nodes should be trusted as delegates. Similarly, in the proposed scheme, the original validator can select a delegated node to perform the role of the original validator on its behalf. If the validator node is subjected to the following conditions, the delegation process will occur. Otherwise, it will be put in blacklist and cannot get the chance to join this network anymore.

- **Moving away from the network**: If the validator node is moving away from the network, it must elect another delegate node to act as a new validator node. Based on Equation 6, the validator node can determine when it must elect a delegate node.
  (6) n+d<t_range,
  where *n* is the number of neighbors, *d* is the distance between each neighbor, and t_range is the transmission range.
- **The validator node is a very rich node**, meaning it has been the MPR node for a long time and accumulated a very high TV (its trust value is already one, which is the maximum value). Therefore, it should give opportunities to other trusted nodes by delegating the block generation process.
- **The validator node is in power-saving mode**. In this case, the validator node does not have sufficient energy to perform additional tasks.

This is when the voting node for the original validator will receive a reward. When choosing a delegate, the validator node will select the node with the highest TV in its neighborhood. However, to give more chances to its original voter, the validator node will temporarily increase that voter’s TV based on Equations 1 and 3. The TV will be increased to the same value as that of the MPR node. Based on this temporarily increased TV, if the original voter becomes the delegate, the temporary TV will be given as a reward for performing blockchain processes. Otherwise, it can only get a portion of the reward for voting for the original validator node.

**Figure 7:**
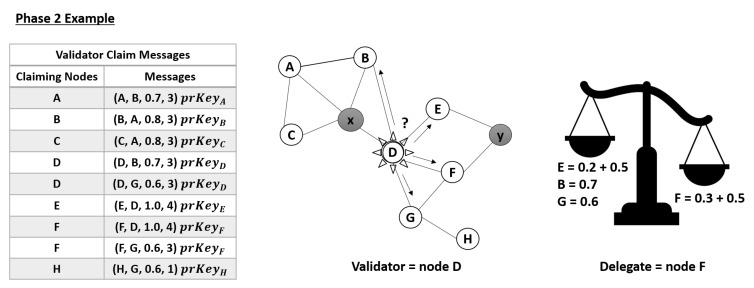
Demonstration of Phase 2.

The demonstration of how to select the validator and how it could delegate to another node is shown in Figure 7. Among the validator claim messages broadcasted by every node in the network, according to node E and F, D has the highest trust value. Even though D is chosen as the validator node, since it is a rich node with trust value ‘’1” it has to perform the delegation process consequently. Among its neighbors, B, E, F and G, it compares their trust values. However, for node E and F, the ones which claimed it as the validator, it compares their value by adding the temporary value, 0.5 to them. Node F with the highest trust value among D’s one-hop neighbors becomes the delegate to create the block.

#### 3.2.3. Phase 3—Transaction Validation & Block Generation

Block transactions update the trust values of all nodes in a network, including malicious nodes. All transactions are sent to the validator node or a delegated node, which will generate a block. This scheme is conducted based on the OLSR protocol, where transactions are disseminated through MPR nodes. Each node n will send an encrypted transaction (n, transaction)prKey*_n_*, where transactions are encrypted by the private key of n.

**Figure 8:**
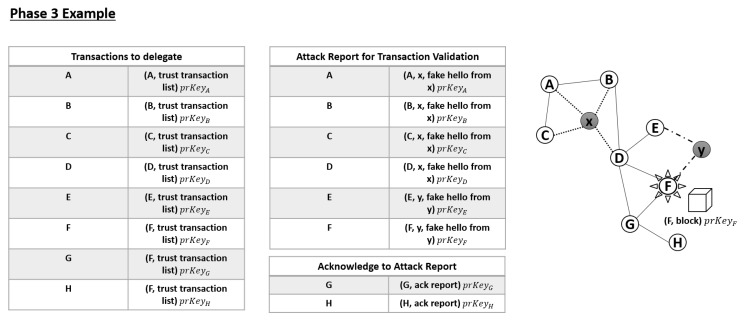
Demonstration of Phase 3.

If a malicious node is revealed by DCFM, it will receive a negative TV and be eliminated from the network. However, one node could erroneously assign a negative value to another node, even if it is not an adversary. To prevent this issue, transactions related to malicious node information must be validated by neighbors before they are sent to the delegate node. The attacker information and any inconsistencies generated by the attacker must be reported through two-hop neighbors because attackers can claim that two-hop neighbors of a victim are their neighbors in NIA. Specifically, a report message (v, x, ReportAttack)prKey*_v_* is sent, where the victim ID (v), attacker ID (x), and ReportAttack (identified inconsistencies) are encrypted by the victim’s private key, as described in Figure 8. This message is sent by piggybacking in hello message until two-hop range since the malicious hello message contains the two-hop neighbors of the victim. If the neighboring nodes agree on the transaction, they will reply (i, AckReport)prKey*_i_* and the transaction will be validated. Since the attacker can also include the fake nodes, to get agreement from all nodes is impossible. Moreover, the node requesting the agreement could also be the attacker which is trying to isolate a good node. Thus, if half of the neighbors included in attacker’s hello agrees with the report, the transaction is validated. In addition, “agree” means that they don’t have connection with that attacker even though it claims that they are its neighbors. Thus, every node is evaluating with the same criteria to agree or not.

Only nodes with TVs above θ will send normal TV transactions that are not attack transactions. The delegate node will count the number of nodes voting for a particular transaction. Based on the count, the delegate will determine the transaction sequence and generate a block. Consequently, the delegate will broadcast the new block (dl, Block)prKey*_d_l* through MPR nodes in the network. When all nodes receive the block, each node replies with a confirmation message (n, BlockAck)prKey*_n_*. If the majority of nodes agree with the new block, each node chains it to a local blockchain.

##### Block Configuration

For creating a block, the information that will be included in the block and the manner in which it will be configured by the delegate node should be specified. In a blockchain system, transactions in the pool are collected as a block and the block is chained through the network. For providing immutability in a blockchain, a hash value (SHA-256 algorithm) that is directly derived from the transaction data is appended to the block. Therefore, even a small change in the data will change the hash value. The hash of the previous block will be included as data in the current block for chaining, meaning a data change in one block can disorganize all blocks in a blockchain. The block hash only accepts a specific format (e.g., a hash signature starting with 10 consecutive zeros). In compliance with this rule, there is a piece of data called a nonce. The nonce value is repeatedly changed until an eligible hash signature is acquired.

**Figure 9:**
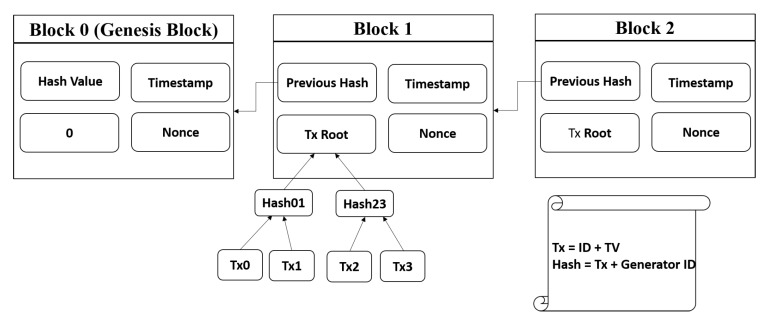
Sample Configuration of a Block.

In a MANET trust blockchain, blocks consist of block transaction data and the metadata described above (timestamp, hash of the transaction, delegate ID, and the nonce). When a transaction is hashed, the transaction generator ID and the TVs proposed by the transaction generator, as well as the delegate ID, will be included to provide non-repudiation for the block transactions proposed by any nodes. When the network is formed, the first block in the blockchain, which is called a “genesis block” (blockchain jargon), is defined with an empty list of transactions. A sample configuration for a block is presented in Figure 9.

#### 3.2.4. Phase 4—Block Maintenance

**Figure 10:**
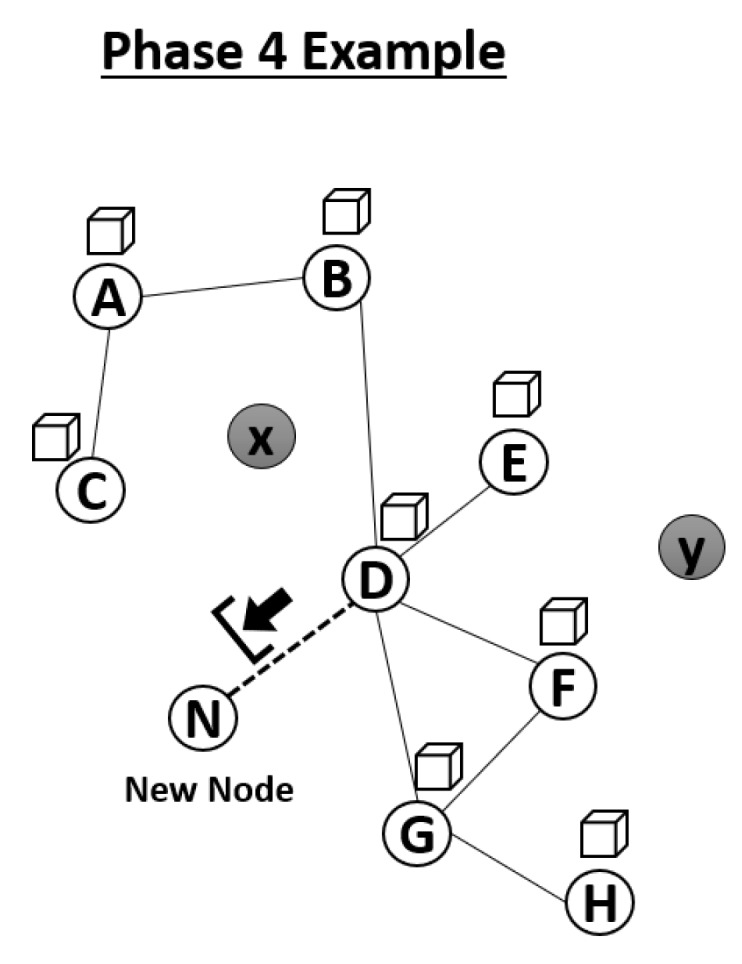
Demonstration of Phase 4.

In a blockchain environment, there are two types of nodes: full nodes, which maintain the blockchain, and light nodes, which largely rely on information from full nodes and do not maintain the entire blockchain. According to the nature of MANETs, we also adopted this concept in our environment. Whenever a new node joins the network, it will have access to the blockchain information. However, a node should initially join the network as a light node, meaning it can simply download the header of the block, as shown in Figure 10. Although a new node will act as a light node immediately after joining the network, it is still able to generate transactions (attacker detection/TV calculation) in the network. The host node of the network will serve as a temporary full node to relay block headers until the new node becomes a full node.

## 4. Performance Evaluation

### 4.1. Simulation Environment

In this section, we present a performance evaluation of the proposed scheme based on the NS-3 Simulator. Because the implementation stack in NS-3 is similar to a real-world implementation, it can be assumed that the simulated scenario will also function properly in a real-world environment. Furthermore, it has built-in implementations of most of the routing protocols in MANETs. Therefore, we used this simulator to implement the proposed scheme to test its feasibility and performance.

We generated a network of 30 nodes with random positions. Sender, node isolation attacker, and victim nodes were predefined and the attacker node was positioned one node away from the victim along the route from the sender node to the destination node. The simulation environment and parameters are summarized in Table 2.

### 4.2. Simulation Results

#### 4.2.1. Effectiveness (Vulnerability Detection)

**Figure 11:**
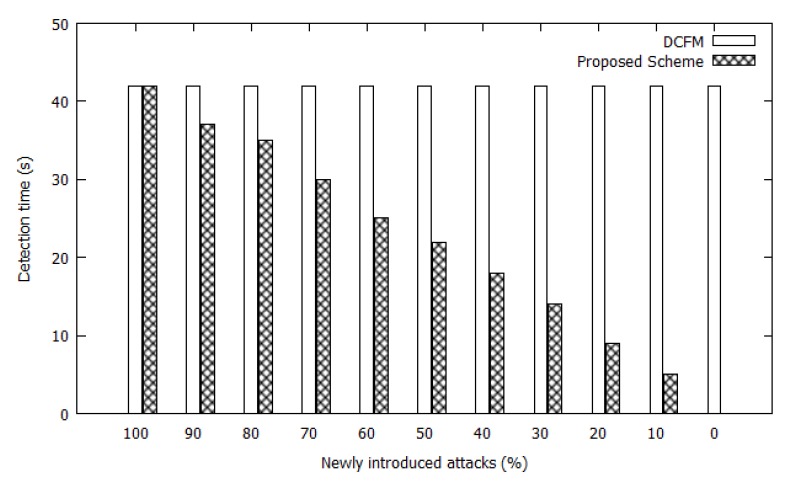
Comparison of the Overall Vulnerability Periods in Networks.

To verify the effectiveness of the proposed scheme, the period of vulnerability to NIAs in a network with the proposed scheme was compared to that in a network with DCFM. Figure 11 presents the overall vulnerability periods based on the time required to detect attacks versus the percentage of newly introduced attacks in the network. According to the standard OLSR protocol, the hello and TC message intervals are two and five seconds, respectively. Therefore, attack detection can take up to five seconds depending on when an attack is launched.

In this simulation, there were ten different potential attacker nodes in the network. We simulated ten sequential attacks at different time intervals. The newly introduced attack ratios varied from 0% to 100%, as shown in Figure 6. According to the DCFM, the nodes are guarding themselves and there is no information being shared between nodes regarding the attacks. Therefore, the nodes must perform the detection process whenever a new attack is introduced. In the proposed scheme, attack information is shared through the network and nodes do not have to repeat attack mitigation tasks that were already performed by other nodes. Therefore, even when the same attacker targets a different victim, a node in the blockchain OLSR scheme is not vulnerable to the attack. In contrast, multiple nodes in the DCFM scheme can be subjected to the attack until it is detected. As a direct result, the vulnerability period of the network with the blockchain OLSR scheme is reduced, as is the ratio of new attackers.

Table 3 shows the percentage of messages received by the victim node when there is an attack and when there is not in the network. In this scenario, we use an attacker node to launch near two different victims. When there is no previous block and also DCFM rules are not applied, it is sure that the adversary had successfully launched the attack and the victim got very few messages. When the rules were using in the absence of block, victim can receive nearly the same percentage of messages as in normal scenario without attack. However, in the third and fourth columns, the percentage of message received is even a little higher than the case of detecting with DCFM. Because of the block distributed in the network, the nodes are directly neglecting the attacker regardless of using DCFM or not. With our proposed scheme, even when the same attacker attacks the different victim, the victim node can still receive the same percentage of messages with no attack scenario. With new attacker in the network, the victim receives the same percentage of messages with DCFM even though it could be a little less than no attack scenario. Therefore, the proposed scheme is proved as an effective solution in terms of security value, too.

#### 4.2.2. Network Overhead

**Figure 12:**
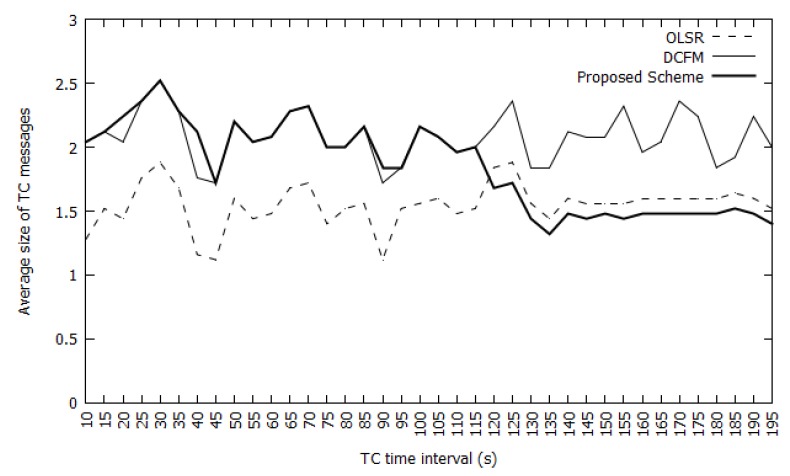
Comparison of Average Overheads (average TC message size).

Figure 12 presents an overhead cost comparison between the OLSR and DCFM schemes. Overhead is measured based on the average TC message size. Our scheme creates a block after detecting an adversary, so it incurs overhead for distributing the block through the network via TC messages. Because adversaries can be detected within five seconds after an attack begins and the TC interval is five seconds in the OLSR scheme, a block will be disseminated within 10 s after an attack. In every second of the TC interval after a malicious node is identified, the overhead will be slightly greater than that of the DCFM scheme.

However, consider a scenario in which six attacks are launched in a network. We launched three different attacks in the first part of the simulation (100 s). The three attackers were detected and three blocks were disseminated through the network. When those attackers attacked different victims during another 100 s period, the detection process was no longer necessary and the attackers were completely ignored by the nodes in the network. Even though six attacks occurred in the network, no additional detection overhead was incurred for the same attackers. Therefore, the overhead of our scheme is significantly reduced for repeated attacks from the same attackers. As shown in Figure 12, the overhead of the proposed scheme is lower than that of the default OLSR scheme because the number of nodes in the network is reduced. This is because previous attackers are ignored in the network. Therefore, it can be assumed that the overhead of the proposed scheme is the same as that of the conventional OLSR scheme.

Additionally, we evaluated the computational complexity for an arbitrary node when it is detecting a malicious node (Table 4). In this scenario, there were six attacks in the simulation. When the victim node detected adversary A, according to the DCFM scheme in Section 2, rule 1 incurred computational complexity of O(n) during the hello interval and rule 2 incurred computational complexity of O($n^{2}$) during the TC interval. The same process is used to detect new attackers in the proposed scheme. Because there were no blocks in the network, our scheme incurred the same complexity. Next, attacker B launched an attack and was detected by a node in both schemes. Subsequently, a different victim was attacked by B again. Attacker B was re-detected by the new victim in DCFM, but not in the proposed scheme. Because there was a block containing attacker B in the network, every other node in the network was aware that B was an adversary. Therefore, they did not need to perform the detection process, which reduced the computational complexity to O(1). Because the DCFM checks if a specific node is an adversary, the necessary computational complexity is O(n) in every hello interval. However, in our scheme, there is no additional complexity incurred for repeated attacks from the same attackers.

#### 4.2.3. Efficiency (Block Generation Latency)

**Figure 13:**
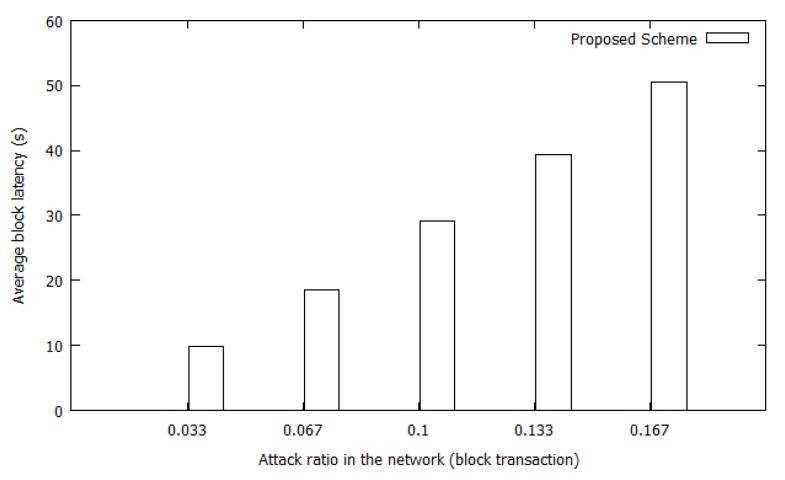
Block Generation Latency Based on Attack Transactions.

In the proposed scheme, the block generation time is much lower than that in other blockchain systems. Ethereum and Bitcoin block times are approximately 15 s and 10 min, respectively. In Figure 13, we demonstrate the lightweight features of the proposed scheme by presenting block latency based on the ratio of attack transactions in the network. Attack ratio is calculated based on Equation 7. To speed up the detection process, hello and TC messages in the OLSR protocol are used to piggyback validation messages and blocks to be transmitted through the network, respectively. For attack transaction validation, the detector node includes attack information in its hello message to receive validation from its two-hop neighbors. As shown in the hello message format (Figure 1), there is a free space called ‘’Reserved” in the hello message, which is used to indicate an attack report. When the detector node includes an attack report for validation, the “Reserved” field is set to a value of one. If the one-hop neighbors of the detector receive a hello message with “Reserved = 1”, this means that the receiver nodes must recognize that there is attack information and relay it to their one-hop neighbors (two-hop neighbors of the detector) to validate the attack report. The neighbors then send a reply message in the next hello message with ‘’Reserved = 1”. Therefore, it costs two hello message intervals (2 * 2 s = 4 s) to validate an attack transaction. In this case, the TVs of any other neighbors in the network will be sent by nodes above the threshold θ to the delegate node via TC messages with ‘’Reserved = 1” to indicate the additional included information. After collecting all transactions, a block will be generated by the delegate and disseminated in the next TC interval.

Therefore, from attack transaction validation to block dissemination, the process requires approximately 10 s, as indicated in the Figure 13. Depending on the time at which an attacker is caught by a victim node, the latency could be a slightly lower or higher than 10 s. For example, if an attack detector node becomes the delegate, the communication time required to relay attack information to the delegate is reduced, leading to even better results in terms of block latency. Additionally, when colluding attacks occur in a network, the block time and transaction ratio are even lower. For example, if two different attackers launch an attack in different places simultaneously, two attack transactions will be included in a block and the effectiveness of the proposed scheme will increase.

(7) $$Attack\_ratio=\frac{number\_of\_attackers\_in\_the\_network}{number\_of\_nodes\_in\_the\_network}$$

## 5. Conclusions

In this paper, we proposed a novel approach for generating distributed trust in MANETs by adopting the blockchain concept. Simulation results demonstrated that distributed trust (i.e., quickly making every node in the network aware of an attacker and any relevant TVs) provides strong network security. Even when an attacker changes their location and attacks different nodes, the network is safe. No information or additional time is lost and overall complexity is reduced. Additionally, based on collaborative detection, each node’s individual responsibility is significantly diminished. The denser the network, the lesser the detection responsibility of each node. Furthermore, by using blockchain-based distributed and tamper-proof access of the trust levels of residents in the network, MANETs can fulfill their system goals such as reliability, scalability, and availability. In future work, we wish to test the feasibility of our proposed scheme with various routing protocols in MANETs. 

## Figures and Tables

**Table 1:** Example Attacks in Different Network Layers.

MANET Layers	Attacks
Application Layer	Malicious code, Repudiation
Transport Layer	Session hijacking, SYN Flooding
Network Layer	Flooding, Blackhole, Geryhole, Wormhole, Link Spoofing, etc.
Data Link Layer	Traffic analysis and monitoring
Physical Layer	Traffic jamming, Eavesdropping

**Table 2:** Simulation Parameters and Values.

Parameters	Values
Simulator	NS-3
Number of nodes	30 mobile nodes
Mobility model	Random Waypoint Mobility Model
Simulation range	1 km × 1 km
Movement speed	3 m/s
Transmission range	250 m
Simulation time	200 s

**Table 3:** Percentages of message Received with DCFM On and Off Before and After Creating Block.

Block	No	No	Yes	Yes
DCFM	Off	On	Off	On
With Attack	21.7	66.45	66.51	66.51
Without Attack	66.51	66.51	66.51	66.51

**Table 4:** Comparison of Computational Complexities for a Node.

-	A	B	B	C	A	C
DCFM	O($n^{2}$)	O($n^{2}$)	O($n^{2}$)	O($n^{2}$)	O($n^{2}$)	O($n^{2}$)
Proposed Scheme	O($n^{2}$)	O($n^{2}$)	O(1)	O($n^{2}$)	O(1)	O(1)
